# Improving Pharmacists’ Targeting of Patients for Medication Review and Deprescription

**DOI:** 10.3390/pharmacy6020032

**Published:** 2018-04-16

**Authors:** Vanessa Marvin, Emily Ward, Barry Jubraj, Mark Bower, Iñaki Bovill

**Affiliations:** 1 Department of Pharmacy, Chelsea and Westminster Hospital NHS Foundation Trust, London SW10 9NH, UK; emily.ward@chelwest.nhs.uk; 2 Medicines Optimisation, NIHR CLAHRC NW London, London SW10 9NH, UK; barry.jubraj@kcl.ac.uk; 3 Institute of Pharmaceutical Science, King’s College London, London SE1 9NH, UK; barry.jubraj@kcl.ac.uk; 4 Department of Oncology, Chelsea and Westminster Hospital NHS Foundation Trust, London SW10 9NH, UK; m.bower@imperial.ac.uk; 5 Department of Elderly Medicine, Chelsea and Westminster Hospital NHS Foundation Trust, London SW10 9NH, UK; inaki.bovill@chelwest.nhs.uk

**Keywords:** deprescribing, medication review, pharmaceutical care, polypharmacy, hospitalisation, frailty, older adults

## Abstract

**Background:** In an acute hospital setting, a multi-disciplinary approach to medication review can improve prescribing and medicine selection in patients with frailty. There is a need for a clear understanding of the roles and responsibilities of pharmacists to ensure that interventions have the greatest impact on patient care. **Aim:** To use a consensus building process to produce guidance for pharmacists to support the identification of patients at risk from their medicines, and to articulate expected actions and escalation processes. **Methods:** A literature search was conducted and evidence used to establish a set of ten scenarios often encountered in hospitalised patients, with six or more possible actions. Four consultant physicians and four senior pharmacists ranked their levels of agreement with the listed actions. The process was redrafted and repeated until consensus was reached and interventions were defined. **Outcome:** Generalised guidance for reviewing older adults’ medicines was developed, alongside escalation processes that should be followed in a specific set of clinical situations. The panel agreed that both pharmacists and physicians have an active role to play in medication review, and face-to-face communication is always preferable to facilitate informed decision making. Only prescribers should deprescribe, however pharmacists who are not also trained as prescribers may temporarily “hold” medications in the best interests of the patient with appropriate documentation and a follow up discussion with the prescribing team. The consensus was that a combination of age, problematic polypharmacy, and the presence of medication-related problems, were the most important factors in the identification of patients who would benefit most from a comprehensive medication review. **Conclusions:** Guidance on the identification of patients on inappropriate medicines, and subsequent pharmacist-led intervention to prompt and promote deprescribing, has been developed for implementation in an acute hospital.

## 1. Introduction

A medicines optimisation project hosted at a 400 bed acute hospital in London began in January 2015 to improve prescribing and adherence in patients with frailty. Interventions included educational updates and medicine safety sessions for doctors and pharmacists in training. The overall aim was to recognise and reduce potentially inappropriate prescriptions (PIPs) in acute hospital settings, and to produce a tool to promote safe deprescribing through targeted review [1,2,3].

Prescribing medicines for in-patients is a routine responsibility held by the majority of medically qualified doctors in the UK, and is a very significant part of the daily duties of junior physicians in particular. In addition, pharmacists, nurses and other allied health professionals may obtain a post-graduate qualification in order to prescribe medicines independently. Independent Prescribing Pharmacists (IPs) are qualified to prescribe in addition to the checking, dispensing, reviewing and advising role of all hospital pharmacists in the UK. Often IPs work within a specialism (e.g., microbiology, neurology, respiratory), as do their senior medical colleagues.

All prescribed medicines, for most UK acute hospital in-patients, are checked for accuracy, reviewed for appropriateness, and reconciled with the patient’s medication history by a pharmacist as soon as possible after admission. The process should start at first contact between the pharmacist and the patient, as suggested by the Royal Pharmaceutical Society [4]. During hospitalisation, medications are reviewed frequently, either (a.) acutely, in response to illness and recovery, or (b.) in a more planned and structured manner, for example during a comprehensive geriatric assessment.

Standards for comprehensive and interim medication reviews were defined at the project initiation (see Box 1), as described in our previous medication review studies [5,6], and used throughout the current work.

Box 1 **Types of Medication Review.**

Comprehensive medication review (adapted from NICE [7])

▸ A structured critical examination by a senior clinician of all current medication, with the objective of reaching an agreement with the patient about optimising treatment and minimising medicines-related problems. With the patient, the reviewer systematically considers the benefits and risks of all medicines, stops or reduces any that are inappropriate, and starts others, taking into account their views and concerns (as well as those of their family members or carers where appropriate).

Interim medication review

▸ In the acute hospital setting, reviews leading to short-term medication changes frequently take place. Medicines are reviewed when a patient presents acutely unwell at hospital, or when their condition deteriorates or improves. Individual medicines may be changed or held because they are considered ‘non-essential’, or currently ‘unnecessary’, or as preparation for a surgical operation. In addition, in these settings, individual medicines are stopped if they are identified as directly contributing to morbidity.

Similarly, our local PIP list was updated from the STOPP criteria of 2015 [8] and those from the NHS Scotland Polypharmacy Guidance in the same year [9]. Additional lines came from other nationally available sources, as cited in our final iteration [1]. 

Local audit revealed a high prevalence of PIPs in falls-risk patients, and at least one inappropriate or unnecessary medicine was found in 19% of inpatients over 70 years old who were assessed as ‘frail’ [6]. Furthermore, nearly a third were taking ten or more regular medicines (a number that appears in our cohort to be associated with more problematic polypharmacy) [10,11]. 

We have previously demonstrated that pharmacists are efficient at recognising PIPs, but less so in timely intervention [6]. For example, alerting the junior doctor to an inappropriate anticholinergic medicine in a patient at risk of falls, can be a misdirected intervention, as our junior doctors are not confident or comfortable at making changes to medication started by a more senior colleague [12]. The process of deprescribing needs a step-wise approach with shared decision-making between clinicians and patients [13], including input from pharmacists. In a review of their impact in medication reviews, it was shown that pharmacists have a positive effect on patient safety through their interventions, although more data is needed [14].

We recognised the need to embed more pro-active pharmacy actions into medication reviews in our hospital practices. For this, we wanted to improve the understanding of roles and shared responsibilities within the multidisciplinary team in targeting vulnerable patients for remedial action, such as dose reduction, holding medication temporarily (hereafter described as ‘HOLD’) and deprescribing.

## 2. Aim

The aim of this phase of our medicines optimisation project was to improve the process of identification by pharmacists of frail patients at risk from their medicines, and to produce guidance on expected actions and escalation processes.

## 3. Objectives

To establish lists of hazardous medicines and combinations to be avoided or reduced in frail individuals. 

To formulate a definitive list of actions for pharmacists when a patient is admitted with, or is at risk of, acquiring a medicines-related problem (MRP).

## 4. Method

An expert panel was convened, comprising four consultant physicians and four senior pharmacists based at acute North West London hospitals, to consider a multidisciplinary approach to reducing inappropriate prescribing in elderly patients. The panel were chosen from colleagues working currently with inpatients and with many years’ experience of the effects of prescription medicines in older adults.

The first consideration was the target age group. Older adults, according to the STOPP/START criteria, [8] are those aged 65 years or older; we have previously used 70 years for our local application of a tool in falls-risk patients [6]. NHS Scotland suggest targeting patients 75 years and older for deprescribing interventions if resources are limited [9]. The panel were asked to consider these target ages i.e., 65, 70 and 75 years; we also tested 85 years and above as a separate older age group. A Medline literature search was carried out, looking for research evidence of who, other than the chronologically ‘old’, are most at risk of medicines related problems (MRPs), and from which medicines. Patients with frailty [15,16], multimorbidity [16,17], polypharmacy [10,11,16,18], on high risk medicines [19,20,21], and high risk combinations [8,9], are described in many of the publications found. Also noted was the increase in risk of adverse drug reactions if a pharmacist had not seen and screened a patient’s medicines on admission, or when there was no system in place for medicines reconciliation [14,16,19]. 

The team met in person to discuss possible ways of identifying frailty through the local electronic prescribing and medicines administration (EPMA) system alerts. Falls risk assessments are documented here and patients are flagged automatically if criteria for falls risk are met. Other ‘alerts’ on the EPMA system, such as dementia and learning disability, were similarly considered by the panel for their usefulness in identifying patients at risk.

PDSA (Plan Do Study Act) cycles were completed in October 2015 and February 2016, yielding a set of ten scenarios that reflect the common situations that pharmacists may encounter with medicines. For each scenario, a choice of six or more actions were described.

These scenarios were emailed, and in addition printed and sent to the panel members for their consideration. They were each asked independently to rank their agreement with each of the possible actions on a scale from zero to nine, with zero as ‘fully disagree’, and 9 as ‘fully agree’. The numerical results were analysed, and where all participants scored zero, 1 or 2, these scenarios were rejected. Through further PDSA cycles and a second face to face meeting of the panel where there was variation and therefore no consensus, the scenarios and actions were redrafted.

The panel were asked to rescore the resulting draft, and this was to be repeated until an agreed list of actions was reached. 

## 5. Results

A set of scenarios covering common clinical situations and medicine combinations were finalised through an iterative process and a series of PDSA cycles. From the original ten scenarios, the wording and age limits for intervention were revised and grouped so that guidelines for pharmacists in identifying and following-up patients at risk in four different situations were produced. 

Consensus was reached in the first round on the following guiding principles:

- Only prescribers can deprescribe. Pharmacists who are not also trained as prescribers* can ‘hold’ medicines acutely, if in the best interest of the patient. In all cases, the reason for holding a medicine must be documented on the prescription, whether on paper or the EPMA; immediate verbal contact should be made with the prescriber or senior colleague.
- The ‘Falls Risk flag’ is not to be used as a target or marker for frailty, as it is too broad and includes many young patients in trauma and recovering from orthopaedic procedures. It was originally suggested as a suitable screening tool for frailty. It was later agreed that it would be more sensitive to screen through age plus a PIP, MRPs or polypharmacy.
- Any inpatient who has a learning disability, hearing or visual impairment, or has a limited ability to speak English, may be at additional risk of harm from medicines, and steps should be taken to ensure these risks are minimised and interventions made with these factors in mind.

*In the UK, registered pharmacists can train and qualify as independent prescribers (IPs). All registered pharmacists, with or without the IP qualification, may perform clinical duties including altering prescriptions for clarification (e.g., formulation change, timing of a dose) and withholding medicines for safety reasons. 

Rejected scenarios from round one were:

1. Patient over 64 but less than 85 years old with falls risk.
2. Patient 85 years old or older with falls risk.
3. Patient over 69 but less than 85 years old on six or more medicines.
4. Patient over 69 years old on 10 or more medicines.

Rejected actions from round one were:

“No action needed” was removed as an option.

“Pharmacist should deprescribe or change any inappropriate medicine and leave a note for the doctor” was removed, leaving the favoured action ‘hold’ and ‘alert’ (see final scenarios below).

Consensus was reached in the second round on the remaining six statements:

- Seventy-five years was selected as the age at which elderly care ‘interventions’ should be made on PIPs.
- Polypharmacy was agreed as six or more regular medicines.
- Sixty-five was selected as the age at which intervention is needed for a patient with a current medication-related problem, including confirmed or potential for non-adherence.
- Sixty-five years was agreed as the age at which intervention is needed for a patient on a hazardous combination; the ‘triple whammy’ or ‘sick day’ medicines [9].

Re-worded and merged scenarios from round two were (originally):

1. Patient over 69 years but less than 85 years old on six or more regular medicines.
2. Patient 85 years old or older on 6 or more regular medicines.
3. Patient over 69 years old but less than 85 on a PIP.
4. Patient 85 years old or older on a PIP.

The following guidance on the pharmacist’s role in prompting medication review was then produced:

1. When a patient is identified as at-risk from their medicines by the pharmacy team (often during medicines reconciliation on admission to hospital), the pharmacist should contact the prescriber immediately to discuss next steps and document actions in the patient’s medical record.
2. In all cases where a junior doctor is the prescriber, the pharmacist should communicate directly with them face-to-face, or immediately by telephone, to inform them of ‘holds’ or to prompt them to take appropriate action. A message can be written in addition to, but not instead of, this dialogue, so that there is an opportunity for learning and improving prescribing practice.
3. In complex cases, junior pharmacists should refer to their senior colleagues for advice. If working outside their area of expertise, pharmacists are encouraged to escalate appropriately to specialists.
4. Details of any medication reviews should be included appropriately in the discharge summary written by the doctor, added to and countersigned by the screening/reconciling pharmacist, whether or not any changes were made prior to the patient leaving hospital.
5. Pharmacist actions are recommended in specific situations given below:

N.B. These lists are not intended to be exhaustive. Screening and checking of all prescribed medicines, combinations and interactions in their clinical settings in all age groups, is expected as a standard for clinical pharmacy; this standard underpins our own current work. Please see Box 2 for key terms and definitions.

Box 2 **Key to Terms Used in Scenarios.**

Complex No strict definition, but includes patients with multimorbidity and on medication for long term conditions where polypharmacy may be necessary. Intervention by the pharmacist depends on his/her own level of competence; changes may require input from other professionals and referral to specialists.
Deprescribe The process of safely stopping regular medicines long term through shared decision making. This is an active systematic process of identifying and discontinuing those medicines with unfavourable risk-benefit trade-offs in the context of illness severity, advanced age, agreed care goals and personal preferences. Deprescribing also involves titrating, changing and switching medicines, but is not about denying effective treatments in eligible patients [22]. Hazardous combinations Medicines prescribed together that adversely interact or compound a clinical condition such as acute kidney injury. See the Triple Whammy and Sick Day Guidance specifically [9].
Polypharmacy For the purposes of this study: six or more medicines taken currently and regularly i.e., not including any ‘as required’ in the count.
Hold The temporary cessation of a medicine with a view to further monitoring and review.
MRP Medicine-related problem, encompassing all adverse drug events and reactions, adherence and supply issues (e.g., medicines, or their omission, contributing to bleeding, falls, confusion, metabolic disturbance, constipation).

### 5.1. Scenario 1

Patient is 75 years old or older on 6 or more regular medicines (polypharmacy) or on a PIP [1]. 

Pharmacist should prompt the junior doctor to review medicines and HOLD or DEPRESCRIBE any unnecessary (i.e., no longer indicated). 

Where there is a risk of acute harm, pharmacist should HOLD any inappropriate medicine immediately and alert the doctor at the first opportunity.

Complex cases should be brought to the multidisciplinary team (MDT) lead clinician, or the pharmacist should otherwise alert the registrar or consultant concerned, to the need for them to arrange a comprehensive medication review (See Box 1).

### 5.2. Scenario 2

Patient is 65 years old or older and presents with a medicine-related problem (MRP) 

Pharmacist should HOLD or reduce any medicine causing harm immediately and highlight the situation to the prescriber at the first opportunity.

Pharmacist should prompt the junior doctor to review the patient and make appropriate changes to the prescription, including starting alternatives to the culprit medicine where needed (e.g., paracetamol in place of codeine for analgesia if constipated).

Pharmacist should notify the MDT, the registrar, or the consultant, of the need for a comprehensive medication review. 

### 5.3. Scenario 3

Patient is 65 years old or older on the ‘Triple Whammy’ [9]; a combination of an angiotensin-converting enzyme inhibitor (ACEi) or an angiotensin-II receptor antagonist (ARB,) plus a diuretic, plus a non-steroidal anti-inflammatory drug (NSAID). 

Pharmacist should HOLD the NSAID immediately and alert the prescriber at the first opportunity.

Pharmacist should prompt the junior doctor to review and HOLD or DEPRESCRIBE appropriately.

Pharmacist should notify the MDT, the registrar, or the consultant, of the need for a comprehensive medicines review. 

### 5.4. Scenario 4

Patient is 65 years old or older with dehydration or signs of acute kidney injury (AKI), and is on metformin, or an ACEi, an ARB, a diuretic, or an NSAID (Sick Day Guidance) [9].

Pharmacist should HOLD all ‘sick day’ medicines [19] immediately and alert prescriber at the first opportunity.

Pharmacist should prompt junior doctor to review all medicines and HOLD ‘sick day’ medicine(s) if he/she has not already done so.

Complex cases should be brought to the MDT, or the pharmacist should otherwise alert the registrar or consultant to the need for a comprehensive medication review.

## 6. Discussion

This improvement work was undertaken as part of a wider project, in which we found there was a need to be more active in the acute setting in reducing inappropriate and harmful prescribing for vulnerable adults. We have developed multi-disciplinary agreed guidance for appropriate positive action in order to tackle problematic polypharmacy. These actions include and encourage safe, targeted deprescribing.

It is recognised that pharmacists have an important role in decreasing medication related problems (MRPs), but as suggested by O’Mahoney [23], agreement is often difficult to reach between doctors, pharmacists, nurses and patients, about *how* it should be done and by whom [23]. We have formed our consensus statements to tackle this area of indecision. We encourage clinical pharmacists to be active participants in MDTs in all areas where medications are reviewed for appropriateness, continuation, or deprescription. Deprescribing is ultimately the responsibility of a qualified prescriber, but in addition to this, our expert panel considers that it needs to be undertaken as an integral part of a comprehensive review. To do the task safely there is a need to include the patient and to consider other medications and interactions.

It is clear that specialist clinicians (including independent prescribing pharmacists) must lead the review in their area of expertise, but this should be supported with more active highlighting of medicines eligible for deprescription by general pharmacists [14,18,23,24,25]. We have addressed this in our suggested actions for pharmacists: that is to ‘hold’ culprit medicines and alert the appropriate clinician to review and take further action. 

The pharmacist should also be challenging unjustified prescriptions and performing regular reviews, whilst engaging with patients in conversations about their medicines, including discussions around medication adherence [26,27]. There is a need for this to be done routinely and systematically—as with optimising drug use in line with kidney and liver function [19]—perhaps by applying screening criteria. Several established tools have been successfully applied in practice, such as STOPP [23,25], and the Good Palliative-Geriatric Practice algorithm [28]. Others have been locally developed utilising an EPMA system for communications [24,29]. A 2013 review of the application of such tools suggests that STOPP identified more medications associated with adverse drug events than the Beers criteria [30]. We reviewed current literature and drew on the clinical experience of senior clinicians to produce the list of clinical criteria and medication combinations that are known to cause harm in aged individuals.

In a systematic review of the risk prediction models available for adverse drug reactions in older adults (over 65 years), evidence was found for the efficacy of having systems in place to ensure reliable medicines reconciliation [19]. The Medication Safety Thermometer (Harmfreecare) http://www.safetythermometer.nhs.uk lists fundamentals for safety as: allergy status documented; medicines reconciliation for all medicines by the pharmacy team (started) within 24 hours of admission; no omission of prescribed medicines without valid reason accurately documented [31]. Omitted and delayed medicines were also the subject of a National Patient Safety Alert in the UK [21]. We are reminded that patients are at risk from unintentional omissions, as well as commissions. 

These publications all suggest that a clinical pharmacy medicines’ screening service with allergy and interaction checks and pharmacist-led reliable reconciliation is fundamental to the safety of hospitalised patients. All are locally integrated into the daily ward pharmacy service at the host hospital, to the standard set out by the Royal Pharmaceutical Society [4], and subject to quality improvement initiatives prior to the current project [32].

However, reconciliation and allergy checks do not identify or prevent all inappropriate prescribing [7].

As described by The British Geriatrics Society (BGS), chronological age is a poor predictor of susceptibility to adverse outcomes; biological age is reflected in the degree of frailty. Frailty identifies older adults who are at increased risk of poor outcomes such as cognitive decline, falls and hospitalization [15]. Medicines are poorly tolerated in frail individuals (even more so when individuals are dehydrated [9,33]), where those over 65 years of age are at particular risk of acute kidney injury [33]. Although there are checklists available, frailty is difficult to assess in practice. We have falls and thrombosis risk checklists, but no Frailty checklist in routine use locally. A recent study suggests they, in any case, these may not be a reliable way of identifying patients who are at risk from their medicines [34]. 

Singled out by the BGS as associated with most adverse outcomes in frailty are anticholinergics, long acting benzodiazepines, NSAIDs and polypharmacy [15]. Those most often associated with problems leading to hospitalisation include antiplatelets, anticoagulants, diuretics and ACE inhibitors [35]. The sets of scenarios and the PIP list we used included reference to these.

Medicines in many situations are known to be unsuitable or hazardous for those over 65 [8,9,19], especially if the patient has a long-term condition; yet there is more likelihood of co-morbidity in the elderly [17]. Those with frailty or cognitive decline are more likely to be on a PIP [36].

Where a MRP is the reason for hospitalisation, documentation of the cause in the patient’s record is vital for safe continuity of care. Graabaek and colleagues describe a successful system where MRPs are communicated to the physician via the medical notes on the EPMA in a Denmark hospital [29]. Our expert panel favoured direct verbal communications followed by clear documentation in the patient’s medical notes. This was agreed as the appropriate action, as it allows for continual learning about safe prescribing and medicines use. There is, at present, a paper medical record and an electronic documentation system linked with the EPMA locally. In practice, pharmacists use the EPMA to record actions required and interventions made. Importantly, these actions and notes regarding medication review and changes to medicines must be transcribed onto the discharge summary letter to the General Practitioner and copied to the patient (and community pharmacist in many cases), to ensure continuity of care.

A decade ago, preventable MRPs were identified as accounting for 3.7% of admissions in one study, and antiplatelets, diuretics and NSAIDs accounted for 50% of these [37]. There is no evidence to suggest this is very different currently.

We have compiled from our work a description of patients at greatest risk from their medicines for particular focus by clinical pharmacists, and a list of culprit medicines and combinations most often implicated. From this, we produced the preferred actions, having reached consensus between eight senior clinicians on what those actions should be. Some patients are at immediate risk; others would benefit from a comprehensive review at some point during their hospital stay. We have put systems in place to enable the most appropriate communication to the prescribing team in common clinical situations. This is expected to lead to more comprehensive reviews and deprescribing, as described with our STOPIT work [2,3] and falls patients previously [6].

## 7. Limitations

We selected six or more regular medicines as Polypharmacy. There are many other limits and definitions; therefore, this may not be universally applicable.

We recruited eight participants to our expert panel. As they were selected by the project team from clinicians we know and work with closely, there is likely bias, and the possibility that the consensus is based on local custom and practice rather than real expertise.

The hospital’s electronic prescribing system is unique, with in-built decision support (again, developed locally), and may not be applicable to areas with paper prescription charts for inpatients or other EPMA support.

## 8. Conclusions

An iterative, scenario-led ranking process achieved consensus on clinical parameters to target at-risk inpatients for medicines optimisation. This targeting strategy was combined with the development of pharmacist-led intervention guidance, including who should review medication, and how urgent actions should be escalated. These interventions are to be embedded into local clinical pharmacy practice. We believe there is scope for a wider application to include care homes, outpatients and other non-acute settings.
